# Ultrasound-guided percutaneous release procedures in the transverse carpal ligament by acupotomy: A cadaveric study

**DOI:** 10.3389/fsurg.2022.906109

**Published:** 2023-01-06

**Authors:** Qiaoyin Zhou, Yifeng Shen, Xinyue Zhu, Xiaojie Sun, Zuyun Qiu, Shiliang Li, Weiguang Zhang

**Affiliations:** ^1^ College of Traditional Chinese Medicine, Fujian University of Traditional Chinese Medicine, Fuzhou, China; ^2^ Key Laboratory of Orthopedics & Traumatology of Traditional Chinese Medicine and Rehabilitation (FuJian University of TCM), Ministry of Education, Fuzhou, China; ^3^ Department of Acupuncture and Moxibustion, China-Japan Friendship Hospital, Beijing, China; ^4^ Department of Urology, Hospital of Chengdu University of Chinese Medicine, Chengdu, China; ^5^ Department of Acupuncture and Moxibustion, Beijing First Hospital of Integrated Chinese and Western Medicine, Beijing, China; ^6^ Department of Hand and Foot Surgery, Beijing University of Chinese Medicine Third Affiliated Hospital, Beijing, China; ^7^ Traditional Chinese Medicine Orthopedics, Beijing Jishuitan Hospital, Beijing, China; ^8^ Health Science Center, Peking University, Beijing, China

**Keywords:** ultrasound guidance, transverse carpal ligament, acupotomy, carpal tunnel syndrome, anatomy

## Abstract

**Objective:**

This study aimed to determine the safety and accuracy of ultrasound-guided acupotomy percutaneous loosening of the transverse carpal ligament.

**Methods:**

The 100 upper limb specimens were equally divided into the ultrasound-guided acupotomy group (*U*) and the nonultrasound-guided acupotomy group (*N*). For the *U* group, we simulated ultrasound-guided acupotomy loosening of the transverse carpal ligament in a human specimen, and for the *N* group, we performed the loosening of the transverse carpal ligament through the same approach under nonultrasound-guided conditions. The safety and accuracy of the two methods were compared through measurement.

**Results:**

In the ultrasound-guided group, the injury rate of nerves, blood vessels and tendons caused by needle-knife release was 0%. In the non-ultrasound-guided group, the rate of nerve, blood vessel and tendon damage was 6 percent, 12 percent and 20 percent, respectively. *χ*^2^ test (Fisher exact test) was performed for the nerve and blood vessel damage rates in the two groups (*P_N_* > 0.05, *P_A_* < 0.05), the difference in nerve damage rates was not statistically significant, but the difference in blood vessel damage rates was statistically significant. Pearson's *χ*^2^ test was performed on the tendon injury rates of the two groups (*P_F_* < 0.05), and the difference was statistically significant. In the ultrasound-guided group, the proportion of acupotomy marks greater than or equal to half of the width of the transverse carpal ligament was 86%, and the non-ultrasound-guided group was 36%. The accuracy of the two surgical methods was tested by Pearson's *χ*^2^ test (*P_L_* < 0.05), and the difference was statistically significant. According to the measurement, the ultrasound-guided acupotomy technology had high safety and accuracy.

**Conclusion:**

In this study, we designed a new method for cutting the transverse carpal ligament under ultrasound guidance, which is different from surgery. These results indicate that this is a safe and accurate method of interventional treatment of carpal tunnel syndrome.

## Introduction

Carpal tunnel syndrome (CTS) is the most common compressive neuropathy (1). Its incidence rate in the United States is 6.7%–7.8% (2–4), and the annual medical cost exceeds approximately 2 billion United States dollars, mostly for surgical treatments (5). The cost of acupotomy for carpal tunnel syndrome is low, the curative effect is good, and the patients readily accept it (6–8). Acupotomy is derived from the acupuncture needles of traditional Chinese medicine. Acupotomy is as effective and safe as surgery for mild to moderate carpal tunnel syndrome. The traditional acupotomy treatment of carpal tunnel syndrome cuts and loosens the attachment point of the transverse carpal ligament to achieve a therapeutic effect (9–13).

However, because acupotomy therapy is a closed operation, visual field blindness requires doctors to have high anatomical knowledge, which increases the risk of the treatment. The development of ultrasound technology can overcome this challenge of closed acupotomy surgery (14). The use of an ultrasound-guided acupotomy can provide a clear view of the acupotomy and the surrounding anatomical structures, which greatly improves the safety and effectiveness of the treatment (15).

Therefore, this study used fixed human cadaver specimens to design a new surgical method for cutting the transverse carpal ligament with an acupotomy under the guidance of ultrasound and to verify the rationality of the approach through studies of clinical anatomy. This new technique was compared with the method of loosening the transverse carpal ligament through the same approach under nonultrasound-guided conditions. This study provides a safe and reliable ultrasound-guided acupotomy release technology reference for the clinical treatment of CTS.

## Materials and methods

### General

**Figure 1:**
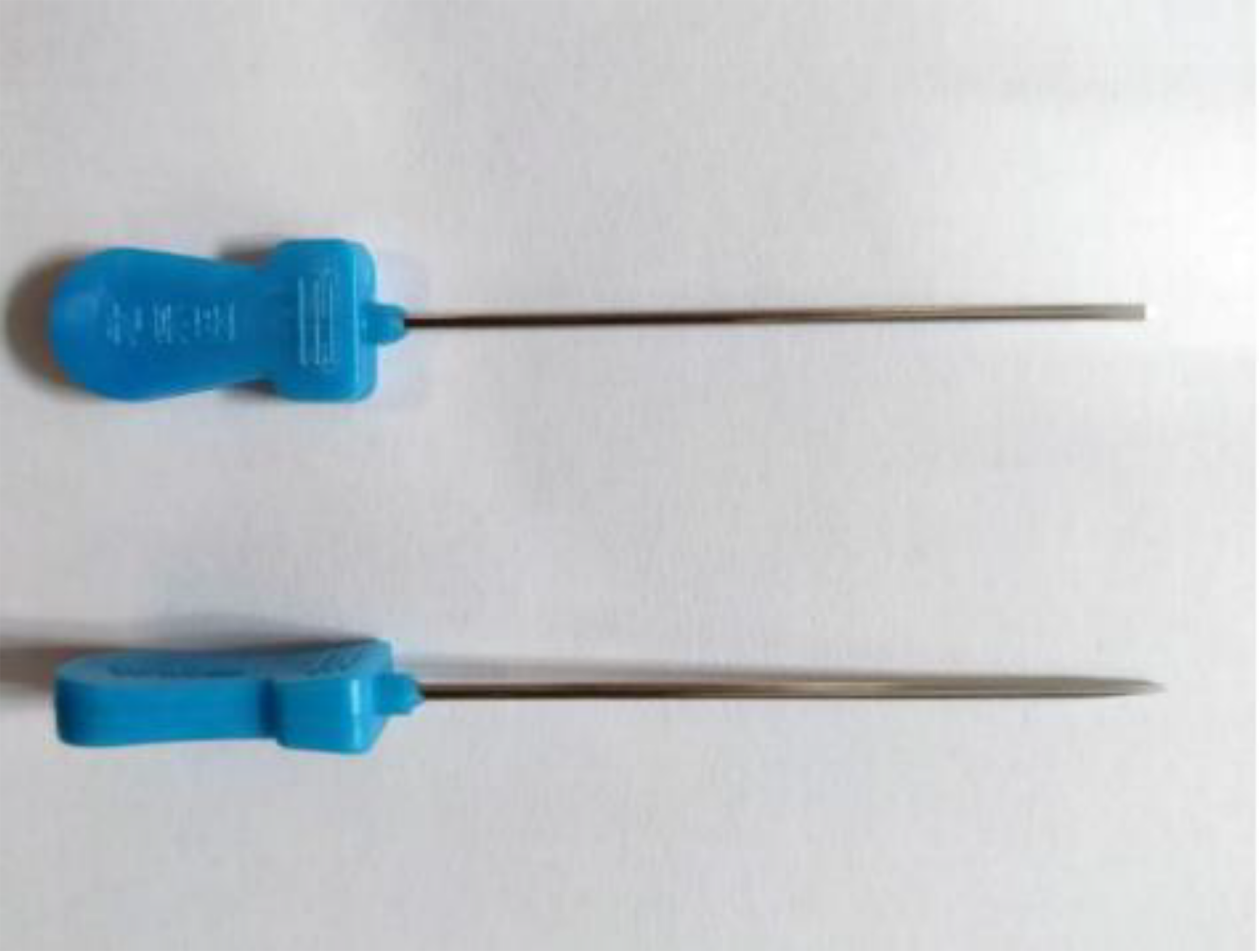
Front and side view of the acupotomy.

Fifty adult specimens (32 men and 18 women) fixed with 10% formalin were selected, aged 52–95 years old, with an average age of 83.36 (SD 8.02) years and a total of 100 upper limb specimens. All specimens were collected from the body donation center of Peking University School of Basic Medicine. All specimens had no wrist injuries, such as deformities, trauma, or obvious degeneration. All experimental operations were performed by one operator. An ultrasound system (Wisonic Medical Technology Co., Ltd., model: Wisonic-Navi) and a high-frequency linear array ultrasound probe (8–13 MHz) were used. An acupotomy (length: 50 mm, diameter: 1.0 mm) was used in this study. The acupotomy is a miniature surgical instrument consisting of a handle, a needle body and a blade (Figure 1).

### Ultrasound-guided acupotomy lysis technique (*U*)

From 100 upper limb specimens, 50 specimens were selected for the operation of loosening the transverse carpal ligament with acupotomy under ultrasound guidance. First, the operator lie the palm of forearm flat, touched the palmar longus tendon with hand, opened the ulnar edge of the palmar langus tendon 3 mm to the side on the ulnar side and intersected the proximal wrist transverse line at point A. Point B was set at the junction of the middle finger and the ring finger, and then the line AB was connected. The Kaplan line was drawed.

**Figure 2:**
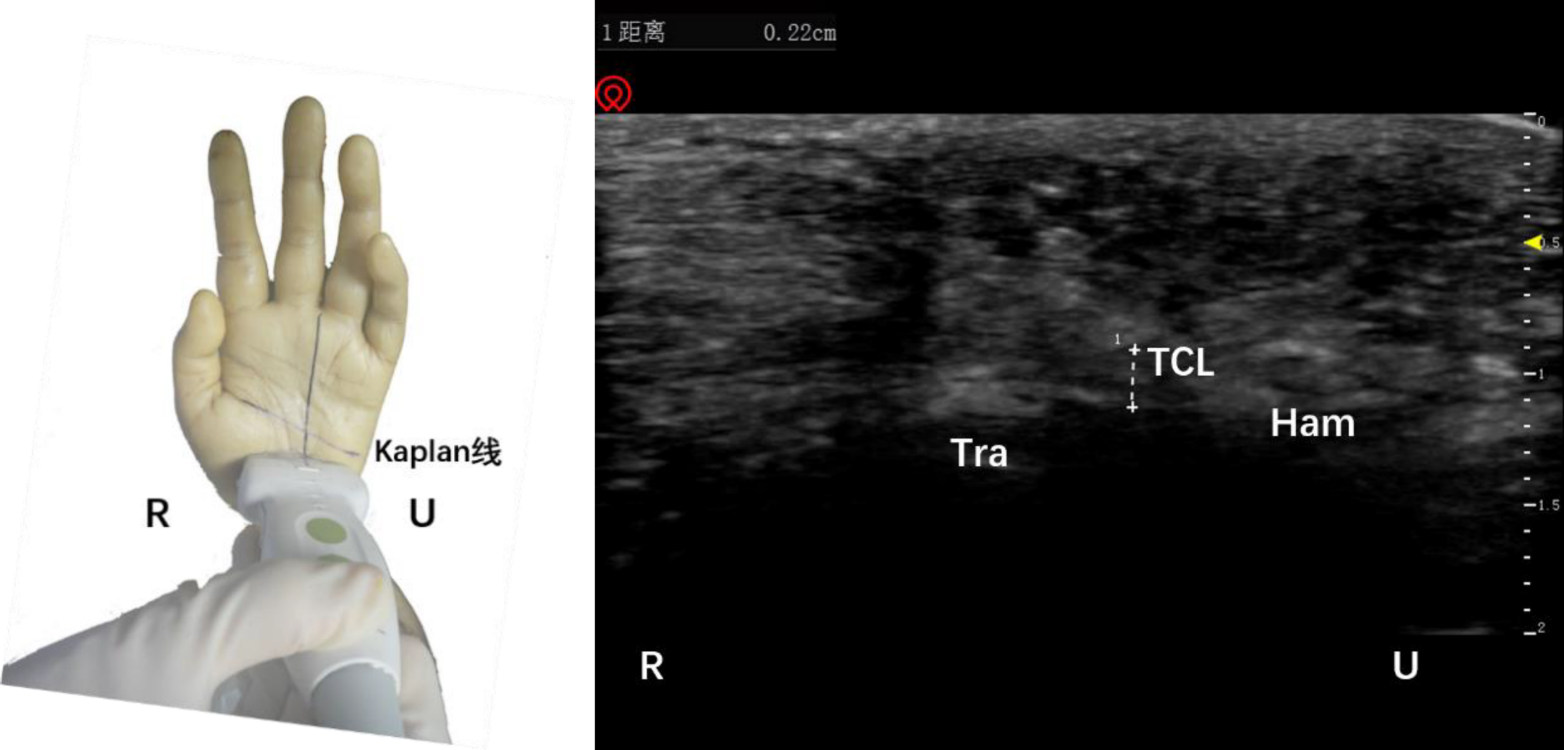
Schematic diagram of probe placement (left) and ultrasound short axis display of the distal boundary of the transverse carpal ligament (right). The plus sign refers to the width of the transverse carpal ligament. Tra, trapezium bone; TCL, transverse carpal ligament; Ham, hamate bone; R, radial; U, ulnar.

After touching the trapezium bone and the Hamate bone, a high-frequency ultrasound probe was used to place the connection line between the two, and the transverse scan was used to identify the distal boundary of the transverse carpal ligament for precise positioning. Note that the midline of the transverse axis of the ultrasound probe was always aligned with AB while keeping the wires overlapping (Figure 2). After marking the surface depth and bottom depth of the transverse carpal ligament in the ultrasound image, the probe was turned to longitudinal scanning. Note that the midline of the longitudinal axis of the ultrasound probe always overlapped line AB.

**Figure 3:**
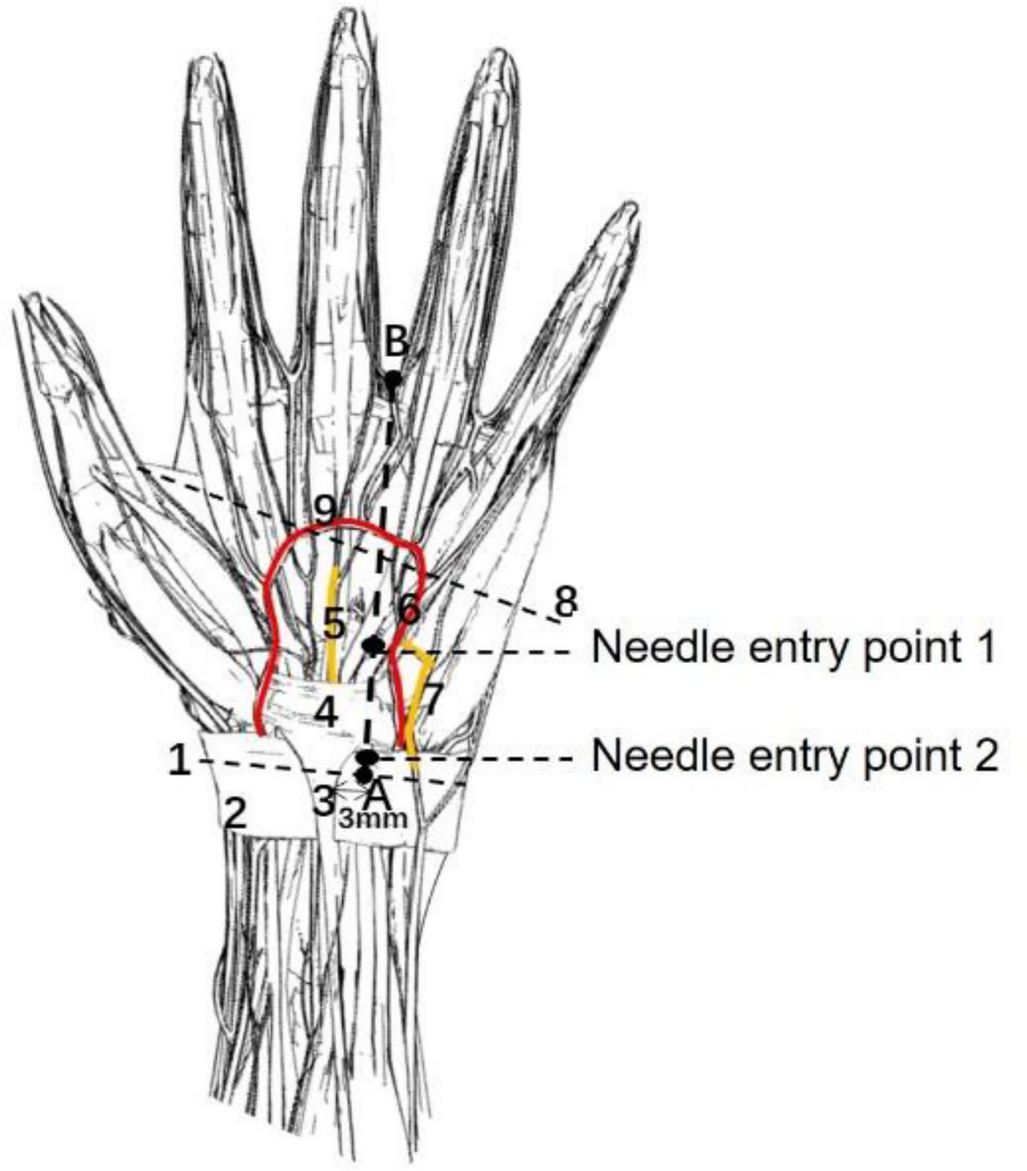
A schematic diagram of the needle entry point and the surrounding anatomical structure. 1, Proximal transverse carpal striae; 2, Superficial transverse palmar ligament; 3, Palmar longus tendon; 4, Transverse carpal ligament; 5, Median nerve; 6, Ulnar artery; 7, Ulnar nerve; 8, Kaplan line; 9, Palmar Superficial arch; (**A**) the intersection of the palmar longus tendon with 3 mm lateral to the ulnar side and the transverse stripes of the proximal wrist; (**B**) the intersection of the ring finger and the middle finger.

**Figure 4:**
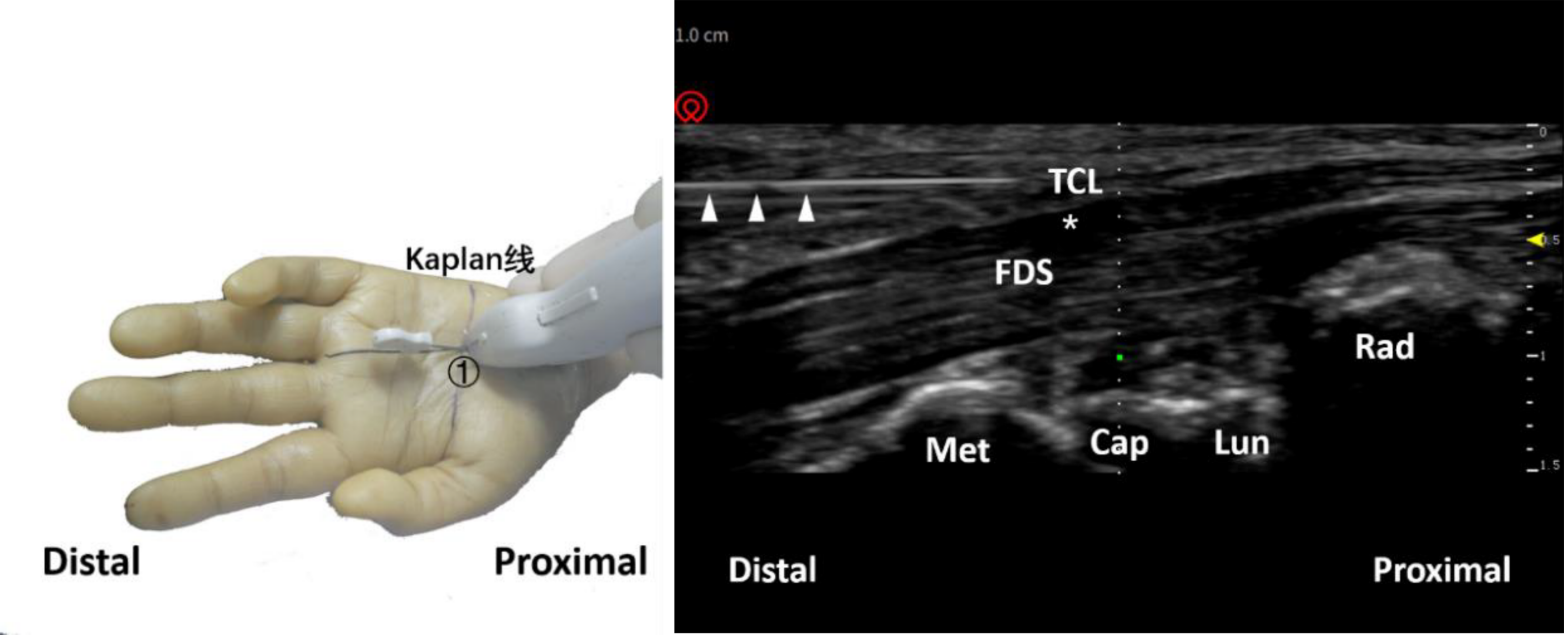
Schematic diagram of probe placement (left) and ultrasound-guided needle knife release diagram (right). The triangle refers to the needle knife position. The asterisk refers to the median nerve. ①, needle entry point 1; TCL, transverse carpal ligament; FDS, flexor digitorum superficialis; Met, metacarpal bone; Cap, capitate bone; Lun, lunate bone; Rad, radius.

Afterward, in-plane needle insertion was performed at the distal end of the transverse carpal ligament approximately 3 mm away from the probe. This was needle insertion point 1 (Figure 3). Note that needle insertion point 1 should not exceed the Kaplan line. When the needle was inserted, the needle body of the acupotomy and the skin fomed an angle of approximately 20°, and the knife surface was held perpendicular to the skin. The ultrasound images showed that when the acupotomy penetrates the skin and reached the surface of the transverse carpal ligament, the transverse carpal ligament needed to be cut 3 times, and cutting was performed. The length of the cut was approximately 12 mm. Note that the vertical depth of the needle tip in the ultrasound image should not exceed the depth of the bottom layer of the transverse carpal ligament previously marked (Figure 4). During the push-cutting process, the touch tissue being cut under the acupotomy could be clearly felt. After the cutting was completed, the needle was removed.

**Figure 5:**
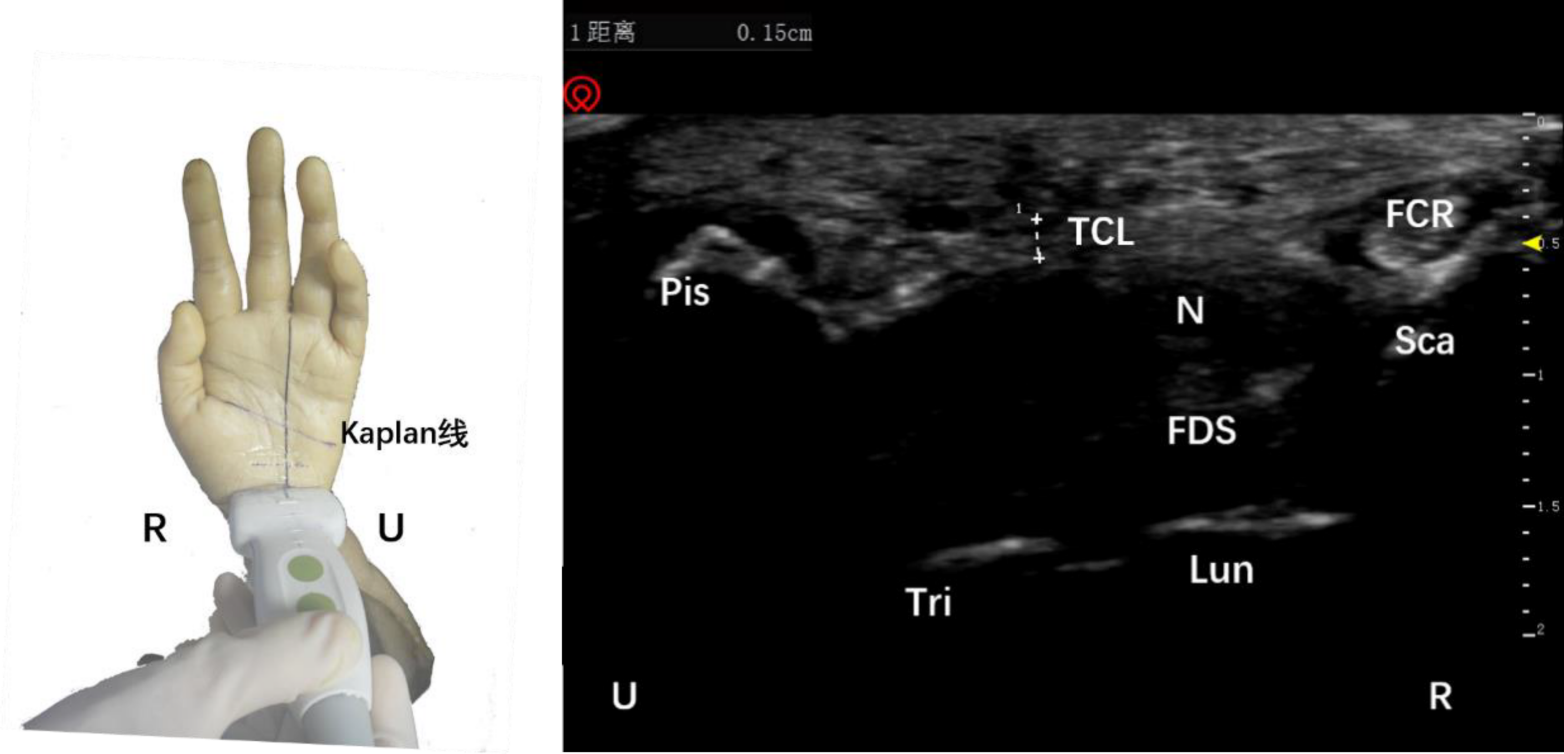
Schematic diagram of probe placement (left) and short-axis ultrasound display of the proximal boundary of the transverse carpal ligament (right). The plus sign refers to the width of the transverse carpal ligament. Pis, pisiform bone; TCL, transverse carpal ligament; FCR, flexor carpi radialis; N, median nerve; FDS, flexor digitorum superficialis; Tri, triquetral bone; Lun, lunate bone; R, radial; U, ulnar.

**Figure 6:**
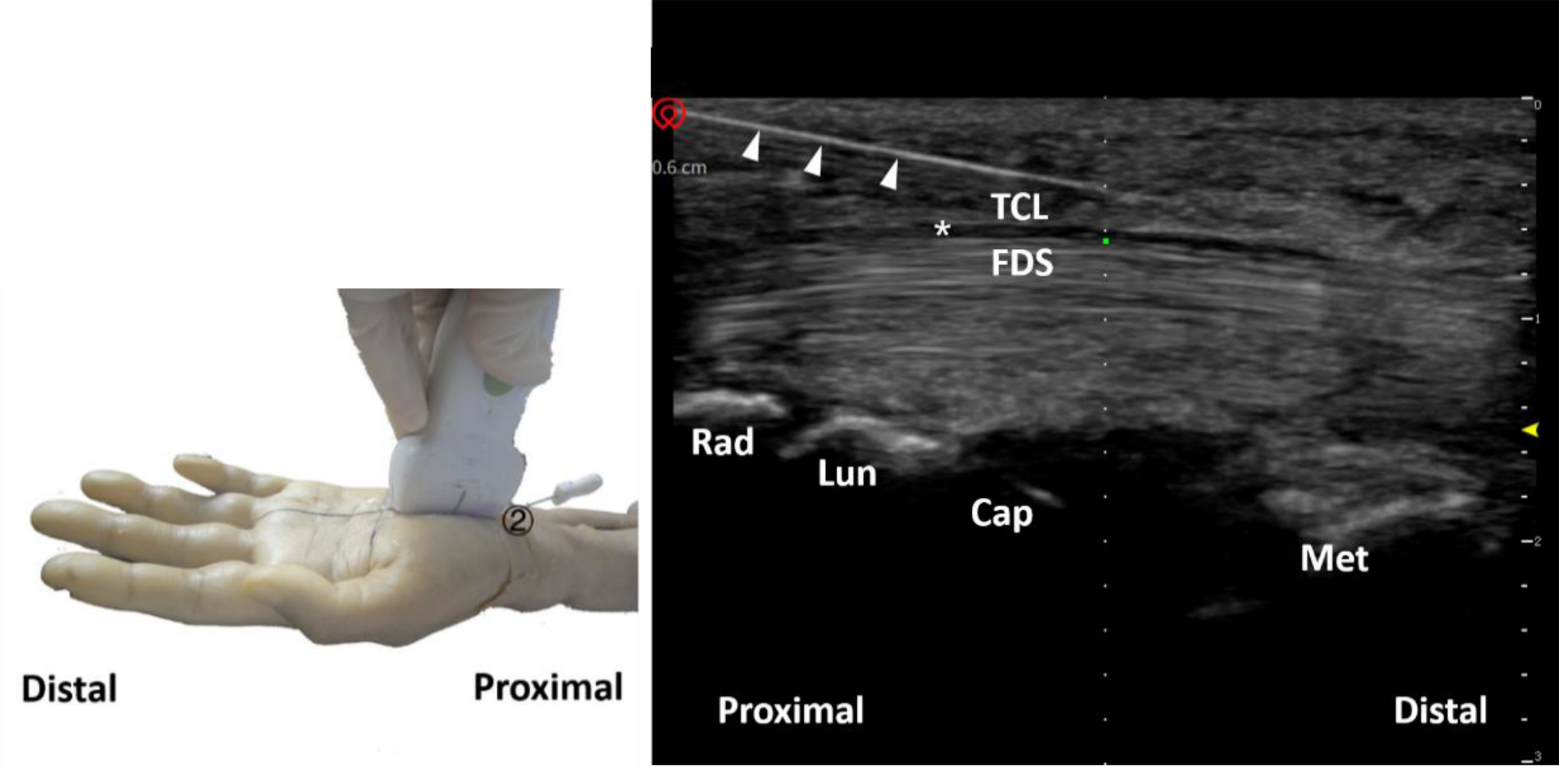
Schematic diagram of probe placement (left) and ultrasound-guided needle knife release diagram (right). The triangle refers to the needle knife position. The asterisk refers to the median nerve. ②, needle entry point 2; TCL, transverse carpal ligament; FDS, flexor digitorum superficialis; Rad, radius; Lun, lunate bone; Cap, capitate bone; Met, metacarpal bone.

**Figure 7:**
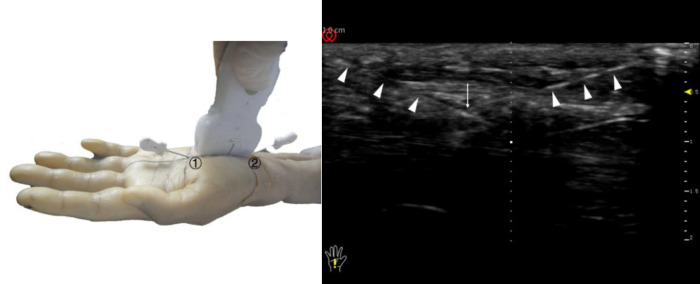
Ultrasound guided needle knife release technique completed (left) and ultrasound long axis display (right). The triangle refers to the needle knife position. The long arrow points to the intersection of the needle points of the needle knife. ①, needle entry point 1; ②, needle entry point 2.

The needle insertion method at needle insertion point 2 was similar to the needle insertion method at needle insertion point 1. After touching the navicular bone and pea bone with hand, the operator used a high-frequency ultrasound probe to place a connecting line between the two, and used a cross-sectional scan to identify the wrist transverse. The proximal boundary of the ligament was marked with the superficial and underlying depths of the transverse carpal ligament in the ultrasound image, and then the probe was turned for longitudinal scanning (Figure 5). In-plane needle insertion was performed at the proximal end of the transverse carpal ligament approximately 3 mm away from the probe. This was needle insertion point 2 (Figure 3). Similarly, under ultrasound guidance, (Figure 6), the transverse carpal ligament was cut 3 times, and the cut length was approximately 12 mm. Finally, the operator removed the needle (Figure 7).

### Non-ultrasound guided acupotomy lysis technique (*N*)

**Figure 8:**
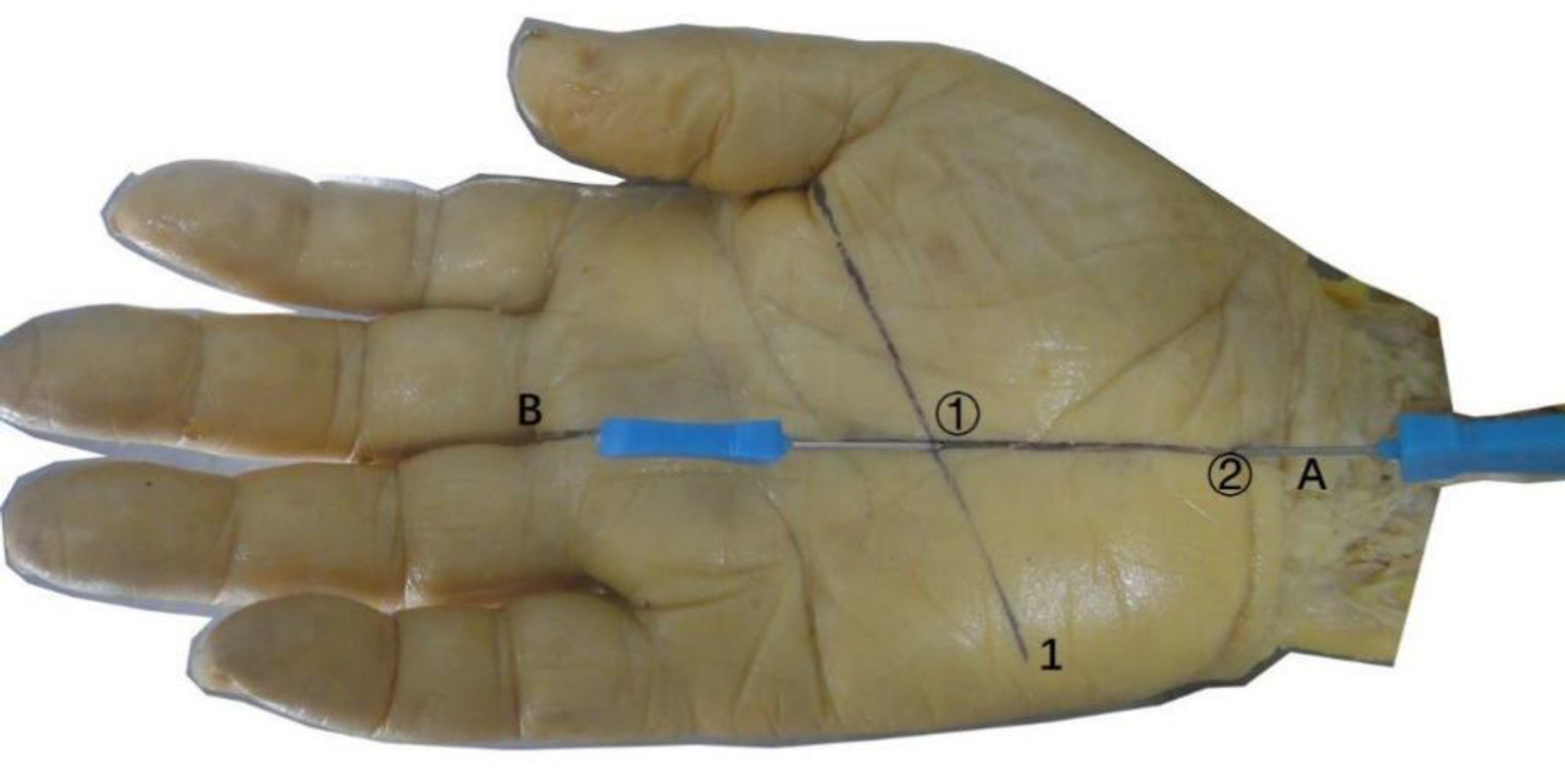
The completed needle insertion technique of the non-ultrasonic guided needle knife release technique. ①, needle entry point 1; ②, needle entry point 2; 1, Kaplan line; (**A**) the intersection point of the palmar longus tendon with 3 mm lateral to the ulnar side and the transverse stripes of the proximal wrist; (**B**) the intersection of the ring finger and the middle finger.

The other 50 cases underwent a procedure using a nonultrasound-guided acupotomy to loosen the transverse carpal ligament. Without ultrasound guidance, the acupotomy was inserted percutaneously into needle insertion point 1 and needle insertion point 2, and when it reached the transverse carpal ligament and the needle tip seemed to touch the tough tissue, the transverse carpal ligament was cut three times. The cutting length is approximately 12 mm, and then the needle was removed (Figure 8).

### Local anatomy of the carpal tunnel acupotome retention site

**Figure 9:**
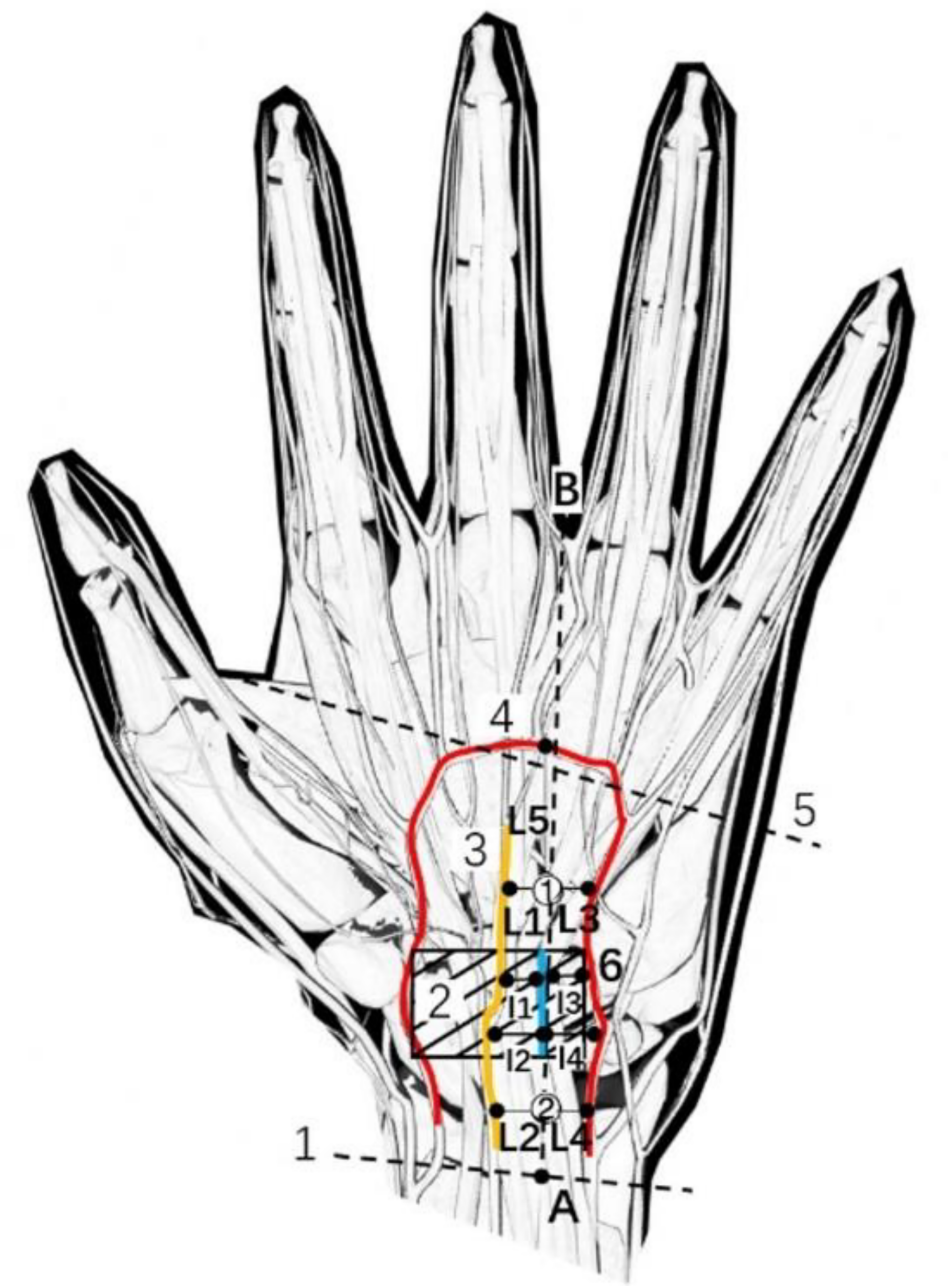
Schematic diagram of measurement indicators. 1, Proximal transverse carpal striae; 2, Transverse carpal ligament; 3, Median nerve; 4, Palmar Superficial arch; 5, Kaplan line; 6, Ulnar artery; ①, needle entry point 1; ②, needle entry point 2; (**A**) the intersection of the palmar longus tendon with 3 mm lateral to the ulnar side and the transverse stripes of the proximal wrist; (**B**) the intersection of the ring finger and the middle finger; L1, the shortest lateral distance between needle insertion point 1 and median nerve; l1, the shortest lateral distance between the cutting traces of the acupotomy at needle insertion point 1 and median nerve; L2, the shortest lateral distance between needle insertion point 2 and median nerve; l2, the shortest lateral distance between the cutting traces of the acupotomy at needle insertion point 2 and median nerve; L3, the shortest lateral distance between needle insertion point 1 and ulnar artery; l3, the shortest lateral distance between the cutting traces of the acupotomy at needle insertion point 1 and ulnar artery; L4, the shortest lateral distance between needle insertion point 2 and ulnar artery; l4, the shortest lateral distance between the cutting traces of the acupotomy at needle insertion point 2 and ulnar artery; L5, the shortest lateral distance between needle insertion point 1 and palmar superficial arch; l5, the shortest lateral distance between the needle tip at needle insertion point 2 and palmar superficial arch.

The shortest lateral distance (L1 and L2, l1 and l2) between needle insertion point 1 and needle insertion point 2 and the cutting traces of the acupotomy from the median nerve were measured on the cadaver, and the median nerve was directly observed to determine whether there were traces of cuts, whether the median nerve was cut, whether the sheath was damaged (none; minor damage: slight scratches on the surface; severe damage: the sheath is completely cut by the acupotomy) (Figure 9).

The shortest lateral distance (L3 and L4, l3 and l4) between the acupotomy cutting marks at needle insertion point 1 and needle insertion point 2 from the ulnar artery was measured, and whether there were cut marks on the ulnar artery was directly observed (Figure 9).

The shortest longitudinal distance between needle entry point 1 and the superficial palmar arch (L5) and the shortest longitudinal distance between the needle tip of needle entry point 2 and the superficial palmar arch (l5) were measured, and whether there were cut marks on the superficial palmar arch was directly observed (Figure 9).

We also directly observed whether there was damage to the superficial flexor tendon (none; minor damage: slight damage to the surface or 10% of the thickness of the injured tendon) and whether there was damage to the flexor tendon sheath (none; minor damage: slight scratches on the surface; serious damage: the tendon sheath was completely cut away by the acupotomy).

The injury rates of the median nerve, blood vessels and superficial flexor tendon were calculated. The calculation method was as follows: injury rate (%) = number of injury cases ÷ total number of cases × 100%.

We directly observed whether the cutting marks of acupotomy entry point 1 and needle entry point 2 were located in the transverse carpal ligament or deviated from the target loosening area and we measured the total length of the cutting marks at acupotomy entry point 1 and needle entry point 2 (*L*) and the width of the transverse carpal ligament (*W*). Calculation of accuracy: accuracy (%) = (L ≥ W/2) number of cases ÷ total number of cases × 100%.

The skin was cut while the needle remained in place, and then the soft tissue was separated layer by layer. We observed the structures, blood vessels and nerves along the puncture path, and took pictures of each layer. The acupotomy's position was observed after TCL exposure. Finally, the TCL was removed to expose the median nerve to observe its integrity.

### Statistical analysis

Measurement data such as anatomical data are expressed as the mean and standard deviation (SD), and count data such as accuracy and injury rate are expressed as percentages (%). The *χ*^2^ test was used for comparisons between groups, and the difference was considered statistically significant at *P *< 0.05.

## Results

### Safety assessment

In this study, the acupotomy entry point and the distances between the acupotomy cutting trace and the blood vessel and nerve were measured (Tables 1, 2). In the *U* group, there were 6 cases of median nerve sheath injury, including 5 cases of minor injury with slight scratches on the surface, and 1 case of severe injury (it was cut by the acupotomy), but no obvious actual median nerve injury was seen. There was no obvious ulnar artery injury and no obvious superficial palmar arch injury. Eight flexor tendon sheaths were injured by acupotomy, including 3 cases of severe injury and 5 cases of minor injury. There was no obvious damage to the superficial flexor tendon.

**Table 1:** Safety measurement of needle entry point.

	Length (x±s) (mm)	Maximum value (mm)	Minimum value (mm)
U-L1	2.40 ± 1.44	7.13	0.77
U-L2	2.27 ± 1.30	6.98	0.67
U-L3	3.50 ± 1.65	7.01	0.91
U-L4	4.38 ± 1.95	9.70	1.51
U-L5	3.01 ± 2.01	7.98	0.79
N-L1	4.01 ± 2.05	9.28	0.00
N-L2	3.55 ± 2.30	12.92	0.00
N-L3	3.31 ± 1.67	6.96	0.00
N-L4	3.81 ± 2.04	9.42	0.00
N-L5	4.18 ± 2.64	9.62	0.00

U, ultrasound-guided needle-knife release technique group; N, non-ultrasound-guided needle-knife release technique group; L1, the shortest lateral distance between needle entry point 1 and the median nerve; L2, the shortest distance between needle entry point 2 and the median nerve Horizontal distance; L3, the shortest horizontal distance from the needle insertion point 1 to the ulnar artery; L4, the shortest horizontal distance from the needle insertion point 2 to the ulnar artery; L5, the shortest longitudinal distance from the needle insertion point 1 to the superficial palmar arch.

**Table 2:** Safety measurement of the cutting trace of the needle knife.

	Length (x±s) (mm)	Maximum value (mm)	Minimum value (mm)
U-l1	2.51 ± 1.35	6.54	0.58
U-l2	2.38 ± 1.23	6.18	0.64
U-l3	3.51 ± 1.94	8.73	0.57
U-l4	4.02 ± 2.01	9.65	0.71
U-l5	10.68 ± 3.95	18.67	3.99
N-l1	3.62 ± 2.03	7.88	0.00
N-l2	3.40 ± 1.92	6.69	0.00
N-l3	2.71 ± 1.68	6.31	0.00
N-l4	2.67 ± 1.34	5.65	0.00
N-l5	11.88 ± 6.27	25.08	0.00

U, ultrasound-guided needle-knife release technique group; N, non-ultrasonic guided needle-knife release technique group; l1, the shortest lateral distance between the cutting trace of needle insertion point 1 and the median nerve; l2, the cutting of needle insertion point 2 The shortest lateral distance between the trace and the median nerve; l3, the shortest lateral distance from the cutting trace of needle entry point 1 to the ulnar artery; l4, the shortest lateral distance from the cutting trace of needle entry point 2 to the ulnar artery; l5, the shortest lateral distance from needle entry point 2 The shortest longitudinal distance between the tip of the needle and the shallow arch of the palm.

In group *N*, there were six cases of median nerve sheath injury, including three cases of minor injury with slight scratches on the surface, and three cases of severe injury (cuts by the acupotomy). There were three cases of median nerve injury, four cases of ulnar artery injury and two cases of superficial palmar arch injury. Sixteen flexor tendon sheaths were injured by the acupotomy, including seven cases of minor injury and nine cases of severe injury. There were 10 cases of superficial flexor tendon injury, including five cases of minor injury and five cases of severe injury.

In the *U* group, there were no nerve, blood vessel, or tendon injuries in any of the 50 specimens, and the injury rate was 0%. In the *N* group, there were three cases of nerve injury, six cases of vascular injury, and 10 cases of superficial flexor tendon injury among the 50 specimens. The damage rates to nerves, blood vessels, and tendons were 6%, 12%, and 20%, respectively (Table 3). The *χ*^2^ test was performed on the injury rate of the two types of surgical nerves and blood vessels, *P*_N _> 0.05, *P*_A _< 0.05. The difference in the nerve injury rate was not statistically significant, but the difference in the vascular injury rate was statistically significant, indicating that in terms of blood vessel safety, ultrasound-guided acupotomy technique is better than the nonultrasonic-guided acupotomy technique. Pearson's *χ*^2^ test was performed on the tendon injury rate between the two groups. The *χ*^2^ value was 11.111, *P*_F _< 0.05, and the difference was statistically significant, indicating that the ultrasound-guided acupuncture technique is superior to nonultrasound-guided acupuncture in terms of tendon safety (Table 4).

**Table 3:** Number and percentage of injuries in the two groups.

	Median nerve sheath (cases/percent)	Median nerve (cases/percent)	Ulnar artery (cases/percent)	Superficial palmar arch (cases/percent)	Total flexor tendon sheath (cases/percent)	Superficial flexor tendon (cases/percentage)
U	6/12%	0/0%	0/0%	0/0%	8/16%	0/0%
N	6/12%	3/6%	4/8%	2/4%	16/32%	10/20%

U, ultrasound guided acupotomy release technique group; N, non-ultrasound guided acupotomy release technique group.

**Table 4:** Comparison of injury rates between the two groups.

	Cases	Nerve	Artery	Tendon	*P_N_*	*P_A_*	*P_F_*
U	50	0%	0%	0%	0.242	0.027	0.001
N	50	6%	12%	20%			

U, ultrasound guided acupotomy release technique group; N, non-ultrasound guided acupotomy release technique group.

### Accuracy assessment

In the *U* group, there were 43 cases with acupotomy cutting marks greater than or equal to half the width of the transverse carpal ligament. There are 18 cases in *N* group. Pearson's *χ*^2^ test was performed on the accuracy of the two surgical procedures, *P*_L _< 0.05, and the difference was statistically significant, indicating that the ultrasound-guided acupuncture technique is better than the nonultrasonic-guided acupuncture technique in terms of accuracy (Table 5).

**Table 5:** Comparison of the accuracy of the two groups.

	Cases	W	L	L ≥ W/2	*χ*^2^	*P_L_*
U	50	22.38 ± 1.53	17.52 ± 4.69	86%	26.272	0.000
N	50	23.25 ± 2.39	11.25 ± 2.53	36%		

U, ultrasound-guided acupotomy technique group; N, non-ultrasonic-guided acupotomy technique group; W, width of transverse carpal ligament; L, total length of acupotomy cutting marks.

### Anatomy of the wrist

**Figure 10:**
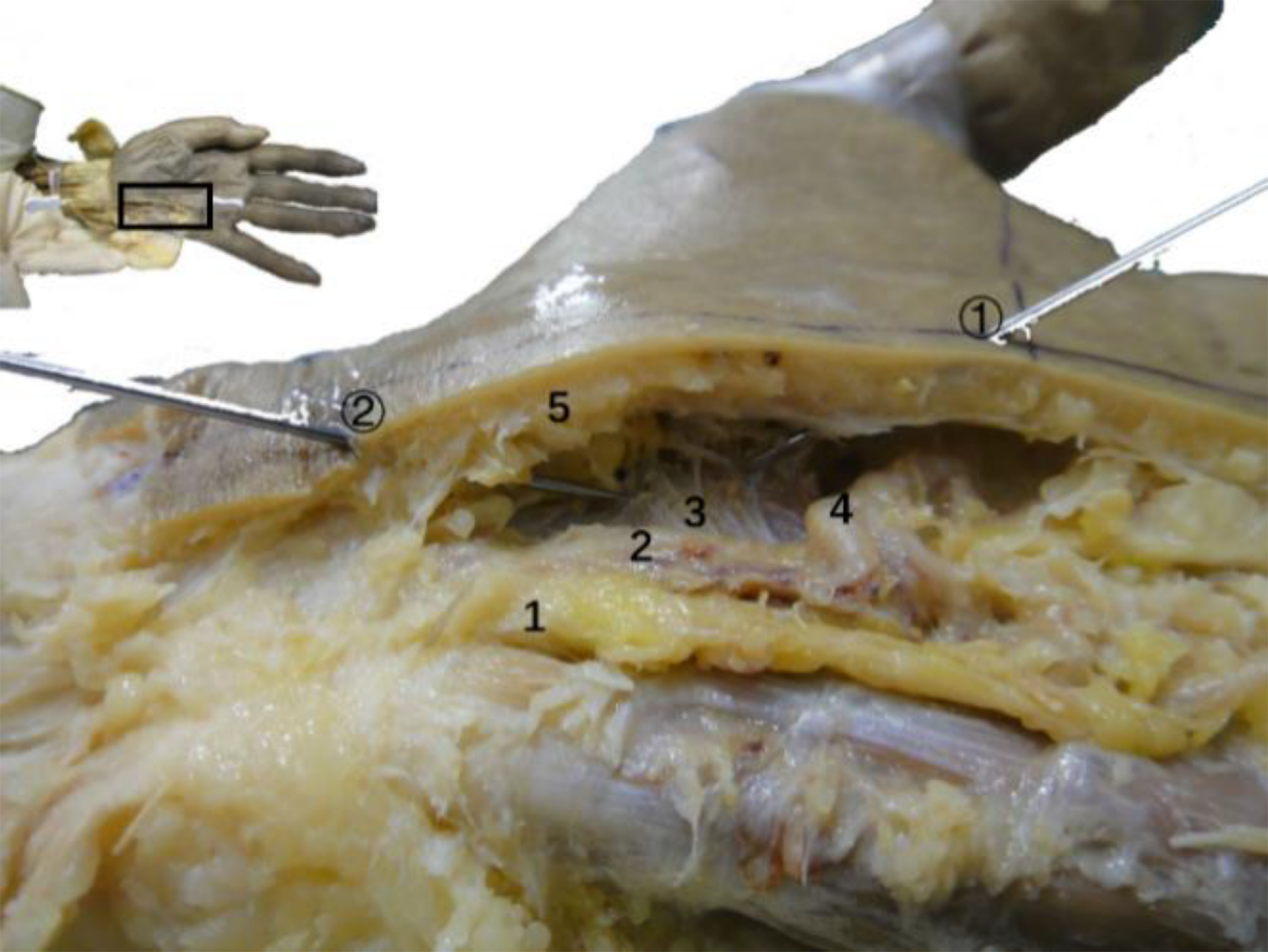
The longitudinal section of the needle-knife release technique guided by ultrasound. 1, ulnar nerve; 2, ulnar artery; 3, transverse carpal ligament; 4, superficial palmar arch; 5, palmar aponeurosis (section); ①, needle entry point 1; ②, needle entry point 2.

**Figure 11:**
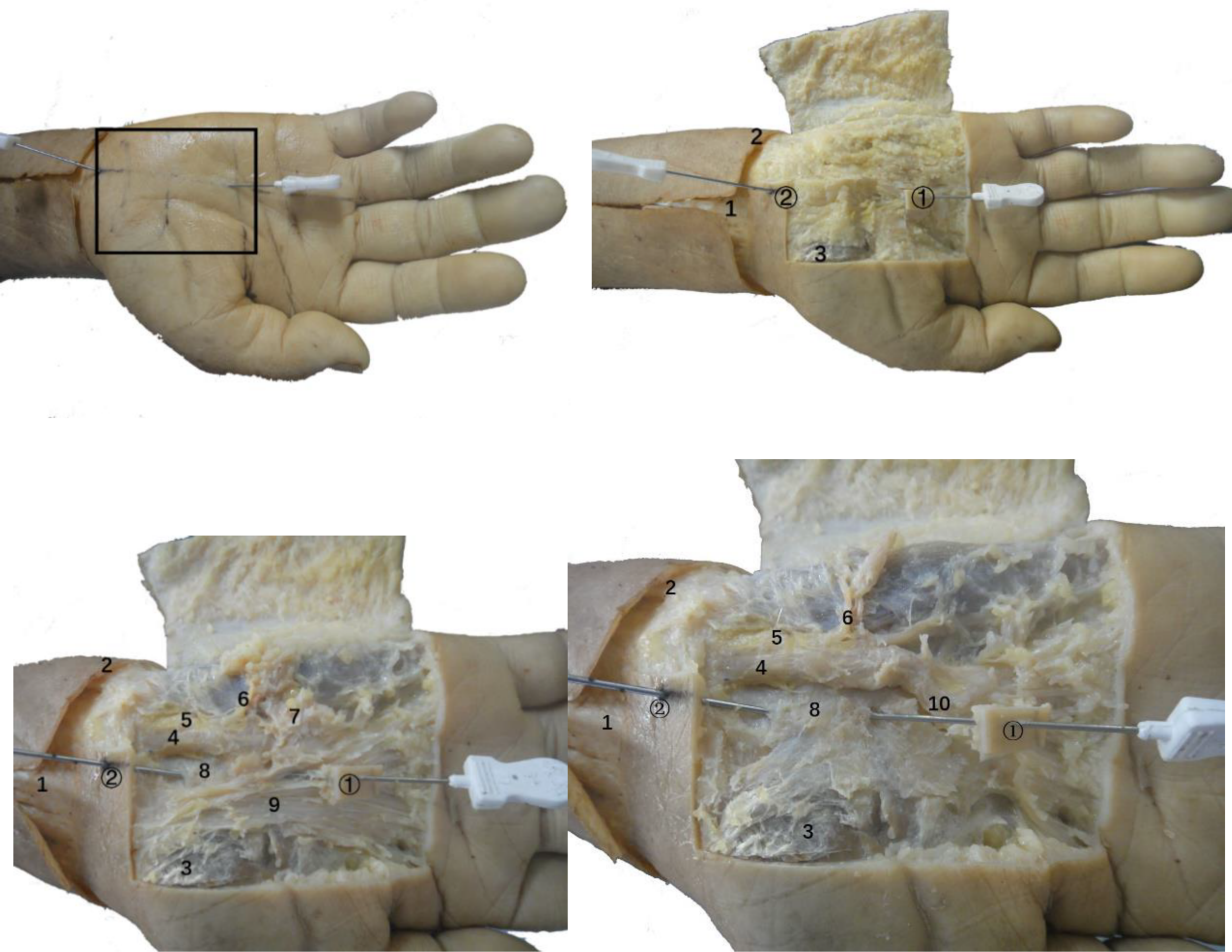
Anatomical view of the needle-knife release technique guided by ultrasound. 1. Palmar longus tendon; 2. Proximal transverse carpal striae; 3. Abductor pollicis brevis; 4. Ulnar artery; 5. Ulnar nerve; 6. Superficial branch of ulnar nerve; 7. Palmaris brevis; 8. Transverse carpal ligament; 9. Palmar aponeurosis; 10. Superficial palmar arch; ①, needle insertion point 1; ②, needle insertion point 2.

**Figure 12:**
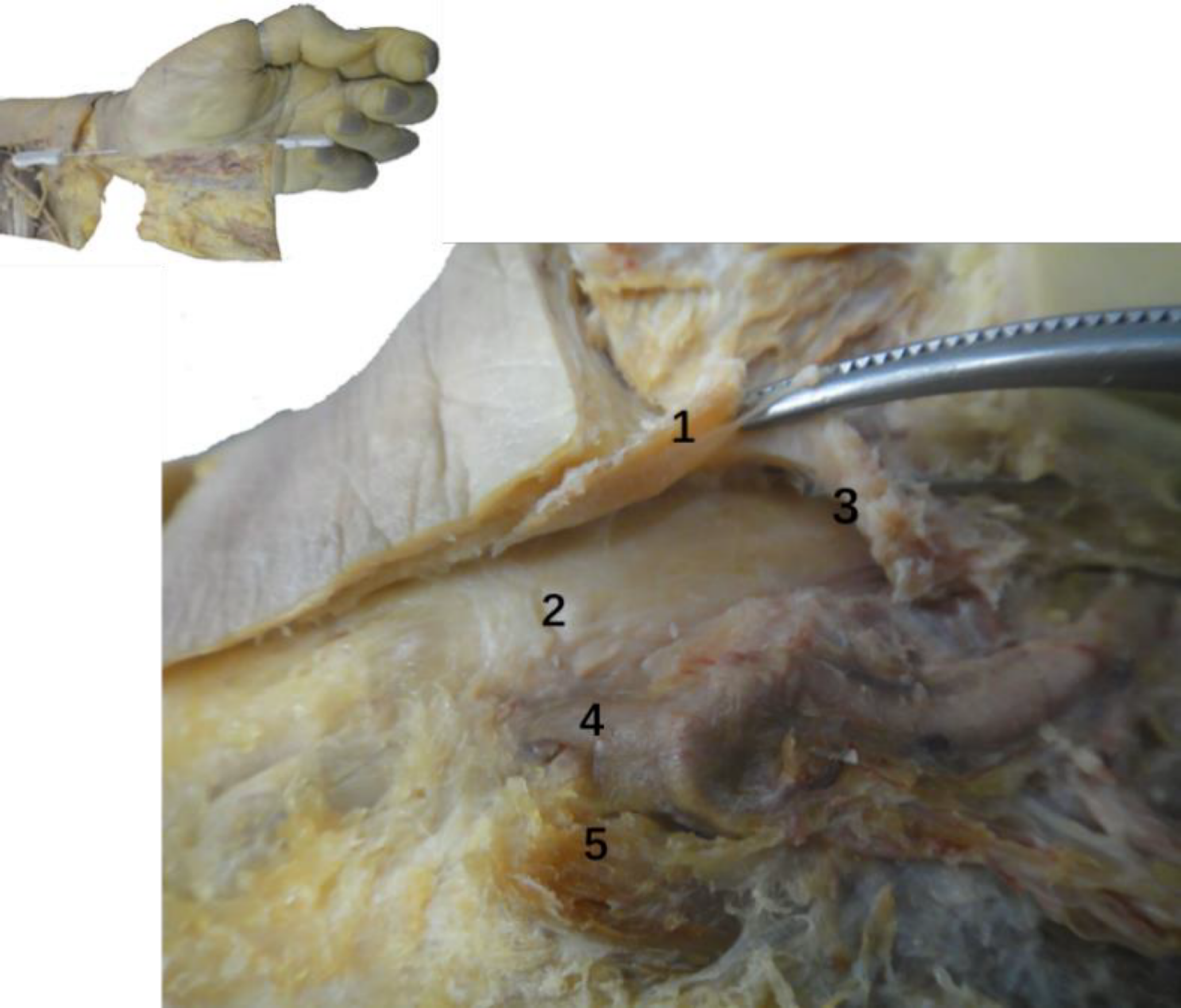
The anatomical diagram of the ultrasound-guided needle knife release. 1. Transverse carpal ligament (turned up); 2. Median nerve; 3. Needle tip; 4. Ulnar artery; 5. Ulnar nerve.

**Figure 13:**
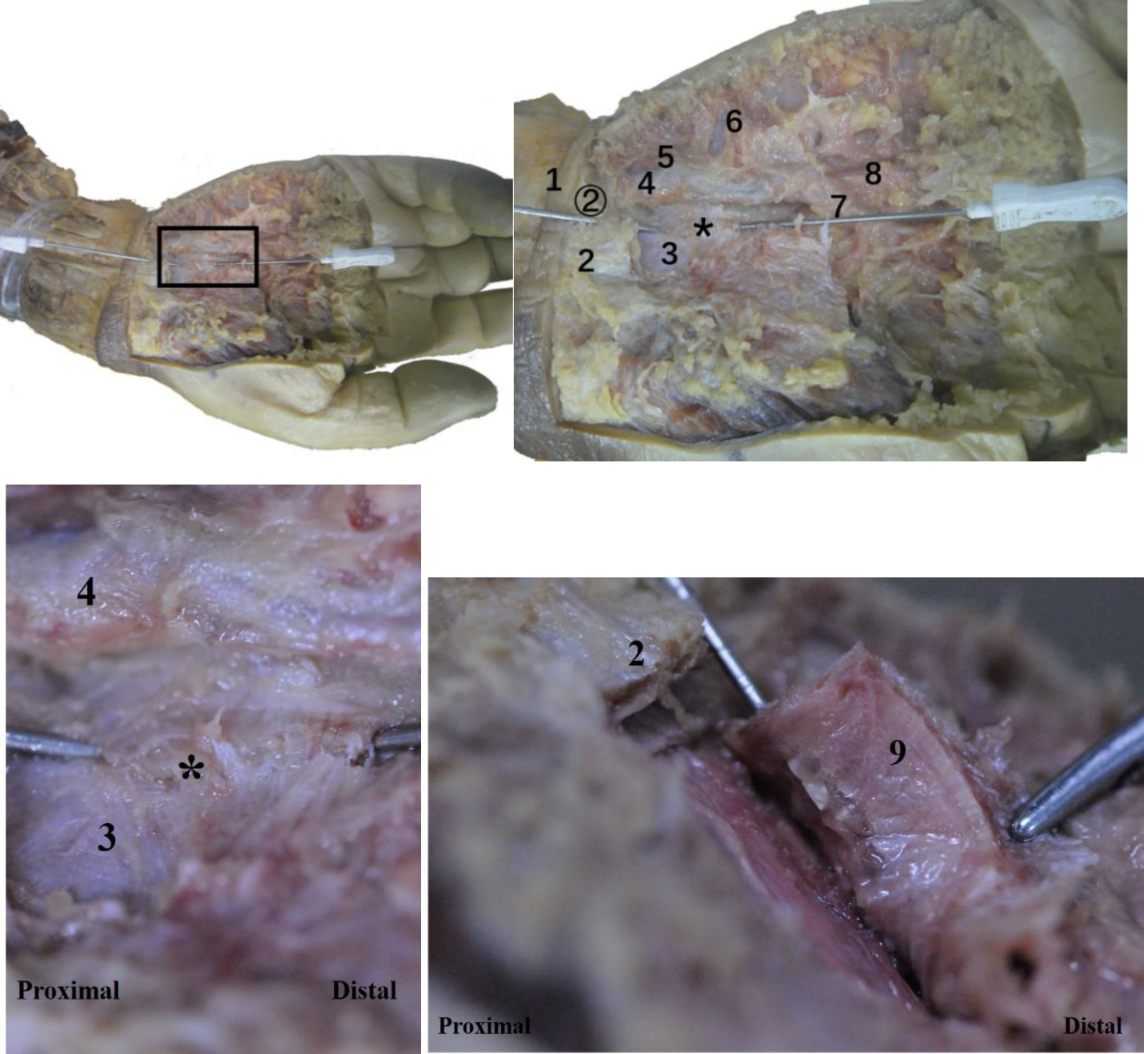
The vertical view (top left, top right) and side view (bottom left, bottom right) of the ultrasonic guided needle knife cutting trace. The asterisk refers to the cutting marks of the needle knife. 1. Proximal transverse carpal striae; 2. Palmar longus tendon connected to the palmar aponeurosis (cut); 3. Transverse carpal ligament; 4. Ulnar artery; 5. Ulnar nerve; 6. Superficial palmar branch of ulnar artery; 7. Palm Superficial arch; 8, common palmar artery; 9, transverse carpal ligament (turned up); ②, needle entry point 2.

Anatomical observation revealed an acupotomy puncture path through the skin, fat, palmar aponeurosis, and transverse carpal ligament in the ultrasound-guided operation (Figure 10). No major blood vessels or nerves were observed in the puncture path. The final needle position was between the median nerve and the ulnar artery (Figure 11). Under direct observation, the exposed median nerve, ulnar artery, and superficial palmar arch were intact (Figure 12), while the transverse carpal ligament had obvious cut marks (Figure 13). This indicates that the cutting method of the ultrasound-guided acupotomy for CTS can cut the transverse carpal ligament and will not damage the main blood vessels or nerves near the puncture path.

**Figure 14:**
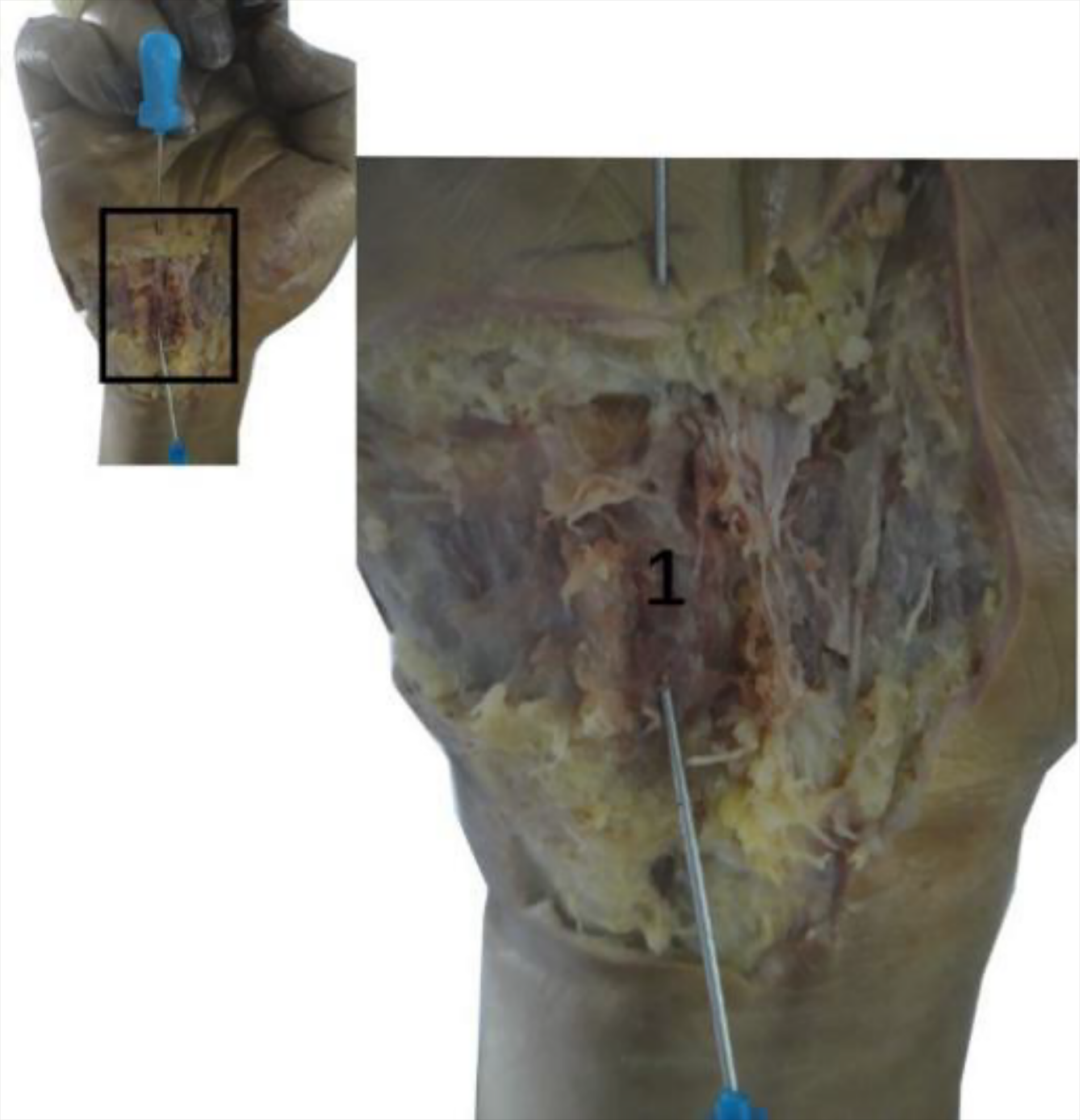
The needle knife penetrated the ulnar artery in the non-ultrasound guided group. 1, Ulnar artery.

**Figure 15:**
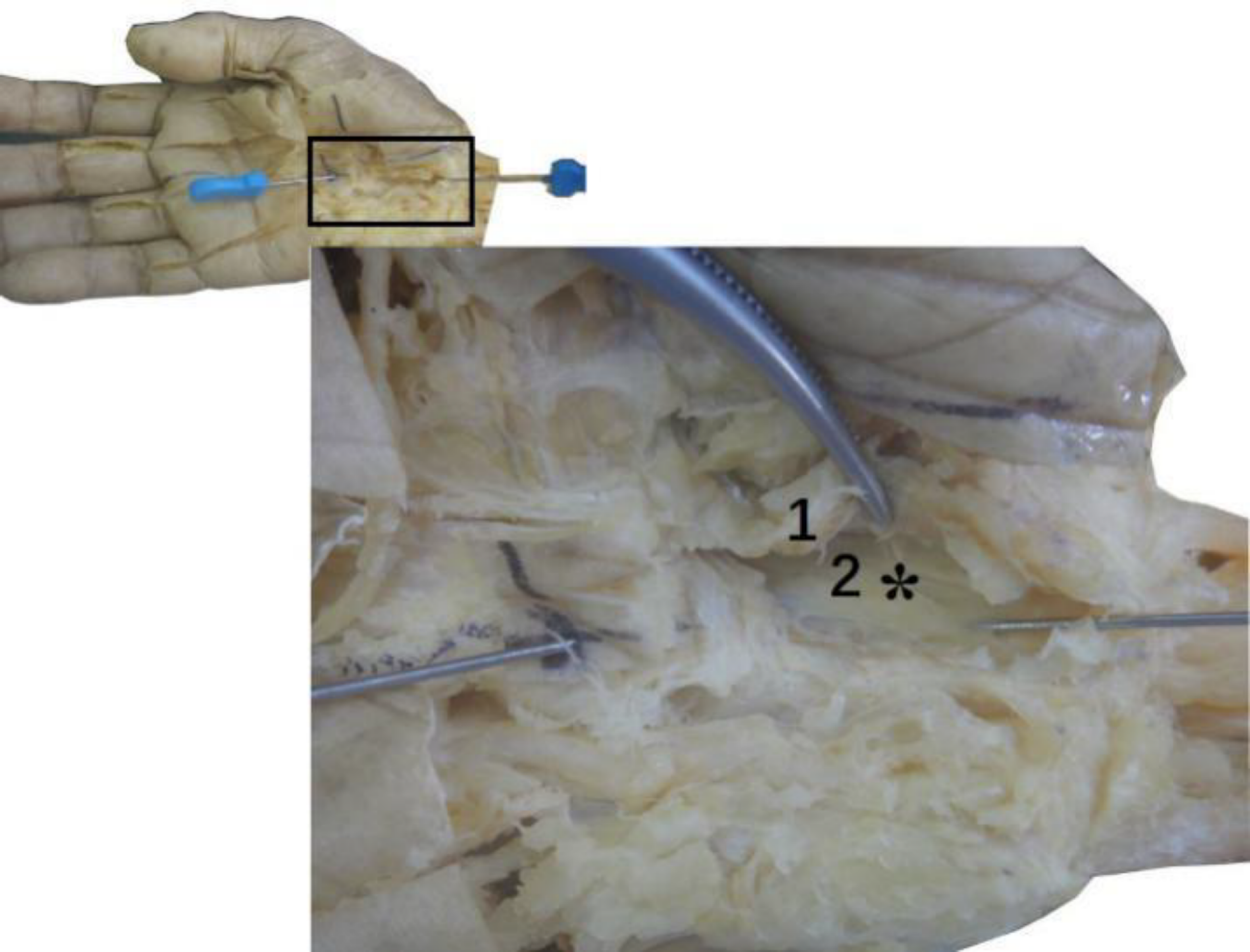
The median nerve was scratched by a needle knife in the non-ultrasound guided group. The asterisk refers to the cutting marks of the needle knife. 1, Transverse carpal ligament (turned up in section); 2, Median nerve.

However, in the nonultrasound-guided group, although the puncture route was the same, the needle body often deviated from the AB line to the left or right under nonvisual conditions, resulting in blood vessel damage (Figure 14) or nerve or tendon damage due to a deep puncture (Figure 15).

## Discussion

### Introduction of the acupotomy

The increased pressure in the carpal tunnel caused by the thickening of the transverse carpal ligament is one of the main causes of carpal tunnel syndrome. Increased pressure in the carpal tunnel can lead to subsynovial connective tissue damage and fibrous hyperplasia, further squeezing the median nerve and causing nerve damage (16). Therefore, loosening of the transverse carpal ligament is an important factor affecting the cure rate of carpal tunnel syndrome (17).

Visualized acupotomy therapy has achieved good clinical effects in CTS patients. The acupotomy is a medical tool used to pierce the human body with a needle to achieve a therapeutic effect. It is similar to a miniature scalpel but its cutting method is contrary to the traditional scalpel. It cuts some fibers of the transverse carpal ligament by pushing and cutting. Generally, in acupotomy, the part to be treated must be cut 3–5 times to form the same cut marks of discontinuous lines. Such surgery can cut the thickened transverse carpal ligament to achieve decompression (Figure 12). The diameter of the acupotomy is usually 0.4–1.2 mm, which is similar to the needle of an ordinary syringe. Therefore, the acupotomy has a shoving and loosening effect during insertion and puncture operations in human tissue so that the human body can repair the damage involved in its use spontaneously, and the possibility of scar formation is extremely low (18).

However, traditional acupotomy treatment is a nonvisual closed operation, lacking objectivity and safety. If acupotomy loosening of the transverse carpal ligament cannot be performed correctly, complications such as vascular and nerve injury will occur during the puncture (19). Therefore, the use of visualization to ensure the safety and effectiveness of acupotomy treatment has become a key issue for its future development. In this study, ultrasound guidance was used to better ensure the safety and feasibility of acupotomy therapy. In addition, ultrasound guidance does not involve radiation.

Ultrasound can provide real-time images that are noninvasive, safe, convenient and flexible. It is therefore suitable for clinical application in acupotomy therapy. However, to the best of our knowledge, there is no prior report of a fixation technique used with ultrasound-guided acupotomy treatment for CTS.

This study designed a surgical procedure that uses ultrasound guidance to visualize the entire acupotomy operation. The image of the tissue targeted by the acupotomy can be displayed by ultrasound. High-frequency ultrasound probes were used to examine carpal tunnel structures, such as the transverse carpal ligament, median nerve, ulnar artery, superficial palmar arch, and the flexor digitorum tendon. In this study, the acupotomy approach was chosen for in-plane needle insertion. This ensures real-time observation of the acupotomy tip and the position of the body during the operation, thereby preventing damage caused by the inability to determine the position of the acupotomy. In addition, ultrasound can realize real-time monitoring of blood flow. However, it cannot be used to monitor blood flow in a corpse. Other studies have shown that ultrasound can be used to monitor the blood flow in the target carpal tunnel to avoid unnecessary injuries during the clinical process.

### Surgical design

The design of this experimental procedure refers to the surgical procedure of carpal tunnel lysis under endoscopy for the treatment of carpal tunnel syndrome (20, 21). To avoid damage to the normal anatomical structure, endoscopic carpal tunnel lysis usually makes anatomical surface markings and drawing the planned incision line before the operation: after marking the palmar longus tendon, follow the palmar longus tendon Draw a 15 mm horizontal line from the distal wrist transverse line to the ulnar side, which is the transverse incision for the endoscope to enter the carpal tunnel; then draw the Kaplan line and intersect it with the longitudinal line drawn along the ulnar side of the middle finger. The mirror usually runs along this longitudinal line of the middle finger and penetrates into the carpal tunnel below the transverse carpal ligament and stops before the Kaplan line. The ultrasound-guided acupotomy treatment in this experiment refers to these two lines.

The Kaplan line is often referred to as the safety marking line for hand surgery. By consulting the literature (22–25), it can be found that there are different anatomical descriptions of the Kaplan line. Two versions can be used as a clinical surgical guide: one is from the line that intersects the fold between the thumb and index finger and the hook bone hook, and the other is to draw a line along the fold that intersects the index finger when the thumb is abducted to the ulnar side, which represents the approximate distal boundary of the transverse carpal ligament range, more than 10 mm away from the shallow palmar arch. The former is a method of drawing Kaplan lines for identifying deep palm structures such as deep palmar arches and deep branches of the ulnar nerve. However, since the surgical procedure studied in this experiment does not involve deep palm structures, it is not discussed here. The ultrasound-guided acupotomy does not go deeply into the carpal tunnel when loosening the transverse carpal ligament and instead pushes and loosens the transverse carpal ligament, so only the superficial structure of the palm is involved.

According to the needs of the operation, the Kaplan line is drawn using the method of “drawing from the vertex of the interfinger fold between the thumb and index finger to the ulnar side of the hand, parallel to the middle crease of the hand” (24, 26). According to an anatomical study of 24 human specimens, the distance between the superficial palmar arch and the extension line of the radial edge of the middle finger is approximately 12.5 mm (27). In, another study that 60 human specimens were dissected to obtain the distance, which was reported to be 10.4 (SD 4) mm (26). Therefore, to avoid damage to the shallow palmar arch, the needle entry point of the acupotomy will need to be below the intersection of the Kaplan line and the AB line. The design of these two needle entry points ensures that the transverse carpal ligament is more fully released and cut, and due to the specific anatomical structure of the carpal tunnel, the median nerve enters and exits the two places of the carpal tunnel, namely, the navicular bone and the pea bone. The space between the large horn and the hook bone is usually the most severe place of nerve entrapment, and it is also the place where most patients have tenderness in clinical practice (28–30). On the other hand, to improve safety, it is necessary to avoid only one needle entry point, since if the needle tip of the acupotomy crosses the Kaplan line it will damages the shallow palmar arch.

### Comparison of ultrasound-guided acupotomy release technique and endoscopic carpal tunnel release

Compared with endoscopic carpal tunnel lysis, the advantage of the ultrasound-guided acupotomy technique is that the acupotomy is released from the surface of the transverse carpal ligament, and the needle entry approach does not pass through dangerous anatomical structures. There was no obvious median nerve or ulnar artery damage in all 50 specimens in the *U* group.

The endoscopic carpal tunnel lysis technique requires the endoscope to pass through the carpal tunnel as a closed compartment, that is, to cut and release the carpal tunnel from the bottom layer of the transverse carpal ligament. However, there is a risk of damaging the median nerve because the carpal tunnel is a cylindrical inelastic cavity, and the endoscope will squeeze the median nerve that is already inflamed and affected by edema in the narrow carpal tunnel (31, 32). The literature (33) has reported iatrogenic injury of the median nerve even if endoscopic surgery is performed accurately. It is worth noting that even experts who perform endoscopic carpal tunnel lysis do not know the threshold pressure that may cause iatrogenic injury of the median nerve during the introduction of the cannula into the diseased carpal tunnel (34).

In this study, if the acupotomy cutting mark (L) was greater than or equal to half of the width of the transverse carpal ligament (W), we recorded it as “accurate” or “effective cut”. This is because we still have doubts about whether the TCL should be completely cut when the patient has CTS for the first time. In the literature (1), it has been shown that complete cutting of the TCL will affect the recovery of the patients' hand function, and some patients will have reduced hand grip strength or hand weakness after surgery.

At the same time, due to the limited visibility of the distal end of the transverse carpal ligament, endoscopic carpal tunnel lysis also leads to incomplete loss of the transverse carpal ligament. Cobb TK and others (27) dissected 50% of 24 human specimens and found incomplete loosening. Some scholars found that this incomplete loosening was approximately 4 mm through anatomical studies, sometimes because the distal end of the transverse carpal ligament did not release, and sometimes there was no release in the middle (22, 27). However, for this incomplete release, studies have shown that the therapeutic effect is no different from that of fully open surgery (35). Cobb TK and others (36) measured the average change in the width of the carpal bone in the palm of the hand after partial and complete release. An anatomical study found that the 4 mm incomplete release of the distal transverse carpal ligament also allowed the expansion of the carpal arch. There is no difference between the results of partial and complete release of the transverse carpal ligament. Therefore, this experimental study also calculated that the incomplete release rate of less than or equal to 4 mm after ultrasound-guided acupotomy release of the transverse carpal ligament was 64%, and cases with complete release accounted for 30%. There were 43 cases where the total degree of release reached more than half of the transverse carpal ligament, accounting for 86%. Of course, whether it has a good effect on the loosening of more than half of the transverse carpal ligament remains to be clinically verified.

The ultrasound-guided acupotomy minimally invasive technique for loosening the transverse carpal ligament is still a conservative treatment and cannot completely replace endoscopic carpal tunnel lysis, but this experimental procedure provides doctors and patients with a choice, that is, as a minimally invasive approach that can be tried first. After all, the time and economic cost of the two are completely different. It takes less time for doctors to learn ultrasound, and it is easier (37, 38). However, endoscopic carpal tunnel lysis has a higher treatment risk, which significantly increases the risk of reversible postoperative injury (32, 39). Brown RA and others (40) therefore advocates for the establishment of effective training programs for surgeons, such as performing simulated surgery training on human specimens. There are also researchers (31) who have conducted relevant studies on whether the results of endoscopic surgery are affected by the proficiency of doctors' surgical skills and found that the difference is statistically significant. Doctors have a learning curve (41, 42), there are high technical requirements and a requirement for anesthesia, requiring the cooperation of anesthesiologists and doctors. In this case, considerable costs will be incurred. The ultrasound-guided acupotomy technique can be performed by a single person, and only one ultrasound device is needed, indicating the requirements for endoscopic equipment are higher (43, 44).

For patients, conservative treatment is more psychologically acceptable and less expensive than surgical treatment. Andreu JL and others (45) and Ucan H and others (46) also recommend conservative treatment first, and only if conservative treatment is ineffective should surgical treatment be attempted. Compared with the ultrasound-guided acupotomy to loosen the transverse carpal ligament, the surgical wound of the endoscopic carpal tunnel lysis is larger, and the risk of complications such as postoperative wound infection is higher (32). Endoscopic surgery requires patients to be hospitalized for observation, and it takes longer for patients to return to work. Ultrasound-guided acupotomy is faster and more convenient. Patients can leave as soon as they are done. They can recover faster and save time and costs for patients. Moreover, patients have greater psychological expectations for surgery and cannot accept surgery-related complications or the results of surgery failure. The literature (47) examined a nationwide legal database and described the most common causes of medical malpractice litigation after CTL surgery. The three most common causes were nerve damage, persistent pain and numbness, and local sympathetic dystrophy.

In addition, ultrasound has an advantage that other tools cannot match, that is, the detection of anatomical variations (34). Studies have shown that if the distance between the median nerve and the ulnar artery is less than 3 mm, endoscopic carpal tunnel surgery is not appropriate to avoid damage to the artery (48, 49). There are also literature studies (34) suggesting that ultrasound or MRI should be used to check whether the patient's wrist has space-occupying lesions before endoscopic surgery and whether it is necessary to change endoscopic surgery to open surgery. Ultrasound can accurately identify peripheral nerves. There have been studies (50) using high-frequency portable ultrasound guidance to carry out some difficult-to-locate extraneural injections of methylene blue on fresh cadaver specimens. The anatomical results revealed that no intraneural nerves were observed under any circumstances. Therefore, whether for acupotomy or endoscopic surgery, preoperative ultrasound-guided neuromapping can help doctors better grasp the disease, thereby minimizing the tissue dissection and operation time.

### Comparison of ultrasound-guided acupotomy lysis technique and non-ultrasound guided acupotomy lysis technique

This experiment designed an ultrasound-guided acupotomy release technique and compared the advantages and disadvantages of this procedure under visual and nonvisual conditions. Anatomical observation results showed that the surgical puncture path does not involve any major blood vessels or nerves. For both safety and accuracy, the ultrasound group was better than the nonultrasound group.

Anatomical research showed that the nonultrasound approach was more dangerous. In 50 specimen experiments, there were 12% and 20% damage rates to blood vessels and tendons, respectively. Clinically, nonultrasound-guided acupotomy treatment is a closed operation, and doctors cannot observe the diseased tissue and cannot effectively identify important and nonimportant tissues. The treatment depends entirely on the doctor's anatomical knowledge and experience, which is likely to cause iatrogenicity. Surgical injuries increase the suffering of patients (24).

However, ultrasound guidance technology makes this problem easy to solve. This study proposes an ultrasound-guided acupotomy to loosen the transverse carpal ligament to treat carpal tunnel syndrome and used 50 fixed specimens of the human body to verify the safety and accuracy of the approach through clinical anatomy. This study provides a safe and reliable reference for the clinical treatment of carpal tunnel syndrome with acupotomy under the guidance of ultrasound. Through experimental research, it can be found that the ultrasound-guided acupotomy to loosen the transverse carpal ligament has unique advantages. Ultrasound exploration can allow doctors to have a clearer and more intuitive understanding of the patient's carpal tunnel and its surrounding anatomy. Ultrasound guidance is also useful during surgical operations. This allows the doctor to more thoroughly loosen the target to be cut. Ultrasound can visualize the transverse carpal ligament, median nerve and acupotomy puncture process, which overcomes the challenge of only relying on the needle feel during the operation.

Therefore, the safer AB line is used to loosen the transverse carpal ligament through ultrasound guidance. Whether it is the needle insertion position or the needle depth, the operation can be performed under visual conditions, which allows for avoidance of the nerves and blood vessels and can also ensure a precise release. This greatly improves the safety and accuracy of acupotomy treatment.

According to the data of this experiment, the clinician can insert the needle at a distance of approximately 38 mm from the proximal transverse crease of the wrist at an angle of approximately 23° from the horizontal line. After entering, distal release of the transverse carpal ligament is performed at a depth of approximately 11 mm. The needle is inserted approximately 3 mm from the horizontal line at approximately 3 mm of the transverse crease of the wrist, and then the proximal end of the transverse carpal ligament is released at a depth of approximately 9 mm. The centerline of the longitudinal axis of the ultrasound probe should overlap the AB line. In practical clinical applications, doctors can appropriately adjust the distance between the needle insertion point and the transverse stripes of the proximal wrist according to the individual conditions of the patient, as long as it is on the AB line and does not exceed the Kaplan line. Before the acupotomy loosening operation, the thickness of the transverse carpal ligament and the distance between the surface layer and the skin should be marked on the horizontal axis; during the operation, attention should be paid to the bottom depth of the transverse carpal ligament marked on the ultrasound image, and the needle tip should not exceed this depth so as not to damage the nerves or tendons.

At the same time, when using ultrasound-guided acupotomy release technology clinically, it is necessary to pay close attention to the ultrasound image and the patient's response. If the position of the tissue and needle tip cannot be displayed during the operation, the acupotomy should not be inserted to a deeper depth blindly to avoid unnecessary damage. We suggest that further studies involving procedures similar to our corpse ultrasound examination are necessary to ensure that the safety of this operation is confirmed before a patient undergoes the operation.

## Data Availability

The original contributions presented in the study are included in the article/Supplementary Material, further inquiries can be directed to the corresponding author/s.

## References

[1] Kim PT Lee HJ Kim TG Jeon IH. Current approaches for carpal tunnel syndrome. Clin Orthop Surg. (2014) 6(3):253–7. 10.4055/cios.2014.6.3.253 25177448 PMC4143510

[2] Dale AM Harris-Adamson C Rempel D Gerr F Hegmann K Silverstein B Prevalence and incidence of carpal tunnel syndrome in US working populations: pooled analysis of six prospective studies. Scand J Work Environ Health. (2013) 39(5):495–505. 10.5271/sjweh.3351 23423472 PMC4042862

[3] Fowler JR Gaughan JP Ilyas AM. The sensitivity and specificity of ultrasound for the diagnosis of carpal tunnel syndrome: a meta-analysis. Clin Orthop Relat Res. (2011) 469:1089–94. 10.1007/s11999-010-1637-5 20963527 PMC3048245

[4] Luckhaupt SE Dahlhamer JM Ward BW Sweeney MH Sestito JP Calvert GM. Prevalence and work-relatedness of carpal tunnel syndrome in the working population, United States, 2010 National Health Interview Survey. Am J Ind Med. (2013) 56(6):615–24. 10.1002/ajim.22048 22495886 PMC4557701

[5] Davis G. Occupation and carpal tunnel syndrome. ANZ J Surg. (2006) 76:1130–1. 10.1111/j.1445-2197.2006.03959.x 17199708

[6] Cong XD. Clinical study of acupuncture combined with local rehabilitation exercise in the treatment of carpal tunnel syndrome. China Medical Innovation. (2021) 18:69–74. 10.3969/j.issn.1674-4985.2021.27.018

[7] Fang YH Wang YL Lin SD. Ultrasound-guided small needle knife release of transverse carpal ligament combined with forearm-related trigger point inactivation for carpal tunnel syndrome. China Medical Innovation. (2021) 18:57–60. 10.3969/j.issn.1674-4985.2021.15.014

[8] Nan L. Observation on the clinical efficacy of acupuncture combined with acupoint injection of methylcobalamin in the treatment of carpal tunnel syndrome. Heilongjiang University of Traditional Chinese Medicine (2021).

[9] Dai M Li KP He NN. Observation on the safety and clinical efficacy of ultrasonic visualization acupuncture technique in the treatment of carpal tunnel syndrome. J Tradit Chin Med. (2020) 38:193–6 +273. CNKI: SUN: ZYHS.0.2020-06-046

[10] Lin HL Huang XH Zhang PP. Observation on the efficacy and safety of ultrasound-guided tooth crochet knife to release transverse carpal ligament in the treatment of carpal tunnel syndrome. China Pract Med. (2019) 14:70–2. CNKI: SUN: ZSSA.0.2019-36-038

[11] Liao AT Li L Liu FS You JY Chen M Fang T A systematic review and meta-analysis of the efficacy of acupuncture in the treatment of carpal tunnel syndrome. J Tradit Chin Med. (2019) 37:2941–7. CNKI: SUN: ZYHS.0.2019-12-030

[12] Zhang XM. Anatomical observation and clinical study of carpal tunnel syndrome treated by needle knife release. Hubei University of Traditional Chinese Medicine (2019).

[13] Zhou QY Shen YF Jia Y Qiu ZY Sun XJ Li SL A clinical anatomical study on the treatment of carpal tunnel syndrome by classical acupuncture. China Orthop Surg. (2020) 33:745–9. CNKI: SUN: ZGGU.0.2020-08-012 10.12200/j.issn.1003-0034.2020.08.012 32875766

[14] Phenix CP Togtema M Pichardo S Zehbe I Curiel L. High intensity focused ultrasound technology, its scope and applications in therapy and drug delivery. J Pharm Pharm Sci. (2014) 17(1):136–53. 10.18433/J3ZP5F 24735765

[15] Visser LH Smidt MH Lee ML. High-resolution sonography versus EMG in the diagnosis of carpal tunnel syndrome. J Neurol Neurosurg Psychiatry. (2008) 79:63–7. 10.1136/jnnp.2007.115337 17470471

[16] Festen-Schrier VJMM Amadio PC. The biomechanics of subsynovial connective tissue in health and its role in carpal tunnel syndrome. J Electromyogr Kinesiol. (2018) 38:232–9. 10.1016/j.jelekin.2017.10.007 29108853 PMC5985810

[17] Yoshii Y Zhao C Henderson J Zhao KD Zobitz ME An KN Effects of carpal tunnel release on the relative motion of tendon, nerve, and subsynovial connective tissue in a human cadaver model. Clin Biomech. (2008) 23(9):1121–7. 10.1016/j.clinbiomech.2008.06.006 PMC2828934 18644662

[18] Zhu X Shen Y Liu Z Gu P Li S Zhang W. Ultrasound-guided percutaneous release procedures in the lumbar ligamentum flavum by acupotomy: a cadaveric study. Evid Based Complement Alternat Med. (2019) 2019:2807901. 10.1155/2019/2807901 31871474 PMC6906825

[19] Li GS Zhang XM Ye WJ. Report of 12 cases of secondary injury treated by small needle knife. J Clin Med. (2000) 4:104–5. CNKI: SUN: JYGZ.0.2000-04-073

[20] Ip WY Sweed TA Fung KK Tipoe GL Pun TS. A new technique of single portal endoscopic carpal tunnel release. Tech Hand Up Extrem Surg. (2012) 16(1):27–9. 10.1097/BTH.0b013e318230db42 22411115

[21] Ruch DS Poehling GG. Endoscopic carpal tunnel release. The agee technique. Hand Clin. (1996) 12:299–303. 10.1016/S0749-0712(21)00312-7 8724581

[22] Cobb TK Cooney WP An KN. Clinical location of hook of hamate: a technical note for endoscopic carpal tunnel release. J Hand Surg Am. (1994) 19:516–8. 10.1016/0363-5023(94)90073-6 8056985

[23] Marmor L. Surgical approaches to the neck, cervical spine and upper extremity. Calif Med. (1966) 105:414. PMCID: PMC1516561

[24] Moran CA. Anatomy of the hand. Phys Ther. (1989) 69:1007–13. 10.1093/ptj/69.12.1007 2685840

[25] Vella JC Hartigan BJ Stern PJ. Kaplan’s cardinal line. J Hand Surg Am. (2006) 31:912–8. 10.1016/j.jhsa.2006.03.009 16843150

[26] Panchal AP Trzeciak MA. The clinical application of Kaplan's cardinal line as a surface marker for the superficial palmar arch. Hand. (2010) 5:155–9. 10.1007/s11552-009-9229-0 19806407 PMC2880680

[27] Cobb TK Knudson GA Cooney WP. The use of topographical landmarks to improve the outcome of agee endoscopic carpal tunnel release. Arthroscopy. (1995) 11:165–72. 10.1016/0749-8063(95)90062-4 7794428

[28] Brooks JJ Schiller JR Allen SD Akelman E. Biomechanical and anatomical consequences of carpal tunnel release. Clin Biomech. (2003) 18(8):685–93. 10.1016/S0268-0033(03)00052-4 12957554

[29] Garcia-Elias M An KN Cooney WP 3rd Linscheid RL Chao EY. Stability of the transverse carpal arch: an experimental study. J Hand Surg Am. (1989) 14(2 Pt 1):277–82. 10.1016/0363-5023(89)90021-X 2703675

[30] Jiang W Haijun X. Progress in biomechanics of carpal tunnel syndrome. Int J Orthop. (2013) 34:58–60. 10.3321/j.issn:1000-0992.2005.04.003

[31] Beck JD Deegan JH Rhoades D Klena JC. Results of endoscopic carpal tunnel release relative to surgeon experience with the agee technique. J Hand Surg Am. (2011) 36(1):61–4. 10.1016/j.jhsa.2010.10.017 21193127

[32] Zuo D Zhou Z Wang H Liao Y Zheng L Hua Y Endoscopic versus open carpal tunnel release for idiopathic carpal tunnel syndrome: a meta-analysis of randomized controlled trials. J Orthop Surg Res. (2015) 10:12. 10.1186/s13018-014-0148-6 25627324 PMC4342088

[33] Uchiyama S Yasutomi T Fukuzawa T Nakagawa H Kamimura M Miyasaka T. Median nerve damage during two-portal endoscopic carpal tunnel release. Clin Neurophysiol. (2004) 115(1):59–63. 10.1016/j.clinph.2003.08.001 14706469

[34] Uchiyama S Itsubo T Nakamura K Kato H Yasutomi T Momose T. Current concepts of carpal tunnel syndrome: pathophysiology, treatment, and evaluation. J Orthop Sci. (2010) 15(1):1–13. 10.1007/s00776-009-1416-x 20151245

[35] Vasiliadis HS Nikolakopoulou A Shrier I Lunn MP Brassington R Scholten RJ Endoscopic and open release similarly safe for the treatment of carpal tunnel syndrome. A systematic review and meta-analysis. PLoS One. (2015) 10(12):e0143683. 10.1371/journal.pone.0143683 26674211 PMC4682940

[36] Cobb TK Cooney WP. Significance of incomplete release of the distal portion of the flexor retinaculum. Implications for endoscopic carpal tunnel surgery. J Hand Surg Br. (1994) 19:283–5. 10.1016/0266-7681(94)90070-1 8077809

[37] Osamura N Zhao C Zobitz ME An KN Amadio PC. Evaluation of the material properties of the subsynovial connective tissue in carpal tunnel syndrome. Clin Biomech. (2007) 22(9):999–1003. 10.1016/j.clinbiomech.2007.07.009 PMC2040304 17822815

[38] Yoshii Y Zhao C Zhao KD Zobitz ME An KN Amadio PC. The effect of wrist position on the relative motion of tendon, nerve, and subsynovial connective tissue within the carpal tunnel in a human cadaver model. J Orthop Res. (2008) 26(8):1153–8. 10.1002/jor.20640 18383182 PMC3901643

[39] Gerritsen AA Uitdehaag BM van Geldere D Scholten RJ de Vet HC Bouter LM. Systematic review of randomized clinical trials of surgical treatment for carpal tunnel syndrome. Br J Surg. (2001) 88(10):1285–95. 10.1046/j.0007-1323.2001.01858.x 11578281

[40] Brown RA Gelberman RH Seiler JG 3rd Abrahamsson SO Weiland AJ Urbaniak JR Carpal tunnel release. A prospective, randomized assessment of open and endoscopic methods. J Bone Joint Surg Am. (1993) 75(9):1265–75. 10.2106/00004623-199309000-00002 8408148

[41] Serra L Panagiotopoulos K Bucciero A Mehrabi FK Pescatore G Santangelo M Endoscopic release in carpal tunnel syndrome: analysis of clinical results in 200 cases. Minim Invasive Neurosurg. (2003) 46(1):11–5. 10.1055/s-2003-37966 12640577

[42] Van Heest A Waters P Simmons B Schwartz JT. A cadaveric study of the single-portal endoscopic carpal tunnel release. J Hand Surg Am. (1995) 20(3):363–6. 10.1016/S0363-5023(05)80088-7 7642909

[43] Saw NL Jones S Shepstone L Meyer M Chapman PG Logan AM. Early outcome and cost-effectiveness of endoscopic versus open carpal tunnel release: a randomized prospective trial. J Hand Surg Br. (2003) 28(5):444–9. 10.1016/S0266-7681(03)00097-4 12954254

[44] Thoma A Veltri K Haines T Duku E. A meta-analysis of randomized controlled trials comparing endoscopic and open carpal tunnel decompression. Plast Reconstr Surg. (2004) 114(5):1137–46. 10.1097/01.PRS.0000135850.37523.D0 15457025

[45] Andreu JL Ly-Pen D. A randomized controlled trial of surgery vs steroid injection for carpal tunnel syndrome. Neurology. (2006) 66(6):955–6; author reply 955–6. 10.1212/01.wnl.0000218667.40662.4d 16567731

[46] Ucan H Yagci I Yilmaz L Yagmurlu F Keskin D Bodur H. Comparison of splinting, splinting plus local steroid injection and open carpal tunnel release outcomes in idiopathic carpal tunnel syndrome. Rheumatol Int. (2006) 27(1):45–51. 10.1007/s00296-006-0163-y 16871409

[47] Gil JA Bokshan S Genovese T Got C Daniels AH. Medical malpractice following carpal tunnel surgery. Orthopedics. (2018) 41(4):e569–71. 10.3928/01477447-20180524-05 29813166

[48] Granata G Caliandro P Pazzaglia C Minciotti I Russo G Martinoli C Prevalence of bifid median nerve at wrist assessed through ultrasound. Neurol Sci. (2011) 32(4):615–8. 10.1007/s10072-011-0582-8 21533564

[49] Kele H Verheggen R Bittermann HJ Reimers CD. The potential value of ultrasonography in the evaluation of carpal tunnel syndrome. Neurology. (2003) 61(3):389–91. 10.1212/01.WNL.0000073101.04845.22 12913205

[50] Gofeld M Bristow SJ Chiu S Kliot M. Preoperative ultrasound-guided mapping of peripheral nerves. J Neurosurg. (2013) 119(3):709–13. 10.3171/2013.5.JNS122243 23829819

